# Treatment of Liver Fibrosis after Hepatitis B with TCM Combined with NAs Evaluated by Noninvasive Diagnostic Methods: A Retrospective Study

**DOI:** 10.1155/2023/5711151

**Published:** 2023-04-25

**Authors:** Li Ying, Zhou Pan, Zhu Lin-Yi, Hong Wan-Er, Wang De-He, Zhang Zhen-Jie, Chu Xi, Wang Yi-Qun, Shen Tian-Bai, Zhang Wei

**Affiliations:** ^1^Department of Hepatology, Longhua Hospital, Affiliated to Shanghai University of Traditional Chinese Medicine, Shanghai, China; ^2^Changhai Community Health Service Center, Yangpu District of Shanghai, Shanghai, China

## Abstract

*Objective*. Chronic hepatitis B liver fibrosis is an important intermediate link in the development of liver cirrhosis. A retrospective cohort study was conducted in Longhua Hospital affiliated to the Shanghai University of Traditional Chinese Medicine in order to prove whether integrated traditional Chinese and Western medicine could improve the incidence of CHB complications and clinical prognosis. There are 130 patients with hepatitis B liver fibrosis (being treated from 2011–2021) included in the study, and the patients were divided into 64 TCM users (NAs combined with TCM) and 66 TCM nonusers (NAs antiviral therapy). The serum noninvasive diagnostic model (APRI, FIB-4) and LSM value were used to classify the stages of fibrosis. The results showed that the LSM value was decreased significantly in TCM users compared with TCM nonusers (40.63% versus 28.79%). Indicators of FIB-4 and APRI of TCM users have improved significantly compared with that of TCM nonusers (32.81% versus 10.61% and 35.94% versus 24.24%). The AST, TBIL, and HBsAg levels in TCM users were lower than those in TCM nonusers, and the HBsAg level was inversely correlated with the CD3+, CD4+, and CD8+ in TCM users. The PLT and spleen thickness of TCM users also were improved considerably. The incidence rate of end-point events (decompensated cirrhosis/liver cancer) in TCM nonusers was higher than that of TCM users (16.67% versus 1.56%). The long course of the disease and a family history of hepatitis B were the risk factors for disease progression, and long-term oral administration of TCM was the protective factor. As a result, the serum noninvasive fibrosis index and imaging parameters in TCM users were lower than those of TCM nonusers. Patients in the treatment of NAs combined with TCM had better prognoses such as a lower HBsAg level, a more stable lymphocyte function, and a lower incidence of end-point events. The present findings suggest the effect of TCM combined with NAs in the treatment of chronic hepatitis B liver fibrosis is better than that of single drug treatment.

## 1. Introduction

Chronic hepatitis B liver fibrosis (hepatitis B liver fibrosis for short) is an abnormal change in the liver structure and/or function caused by long-term chronic stimulation of the hepatitis B virus and excessive proliferation and deposition of extracellular matrix (ECM) components. It is an important intermediate link in the development of liver cirrhosis [1]. More than 2 billion people worldwide are infected with HBV, of which 240 million people develop chronic persistent hepatitis with the risk of continuous progression of liver fibrosis. About 650,000 people die of liver failure, cirrhosis, and hepatocellular carcinoma (HCC) caused by HBV infection every year. The prevalence of HBV in the Chinese general population is relatively high. The prevalence of HBV infection was 6.89% from 2013 to 2017, and there are about 97 million hepatitis B virus carriers in the Chinese general population [1, 2]. Approximately 308,000 people in China die of HBV every year [3]. At present, the percentage of anti-HBV drugs (nucleotide analogues and interferon) reversing liver fibrosis is only 30–40%, and fibrosis may still exist and continue to develop after viral response [4–6]. Liver fibrosis is one of the major global health problems due to the lack of effective treatment. As a reversible stage in the development of chronic hepatitis B (CHB), liver fibrosis plays an important role in preventing the occurrence and development of liver cirrhosis and prolonging the survival of patients with chronic liver disease.

The effect of traditional Chinese medicine (TCM) combined with nucleotide analogues (NAs) in the treatment of chronic hepatitis B liver fibrosis is better than that of single drug treatment in some studies [7]. As the main method to prevent and treat the progress of liver fibrosis, integrated traditional Chinese and Western medicine can improve the incidence of CHB complications and clinical prognosis. However, there is a lack of long-term follow-up of patients with liver fibrosis. Therefore, this study aims to determine the impact of long-term TCM syndrome differentiation combined with NAs antiviral therapy on the clinical efficacy and prognosis of patients with chronic hepatitis B liver fibrosis.

## 2. Methods

### 2.1. Study Subjects

**Figure 1:**
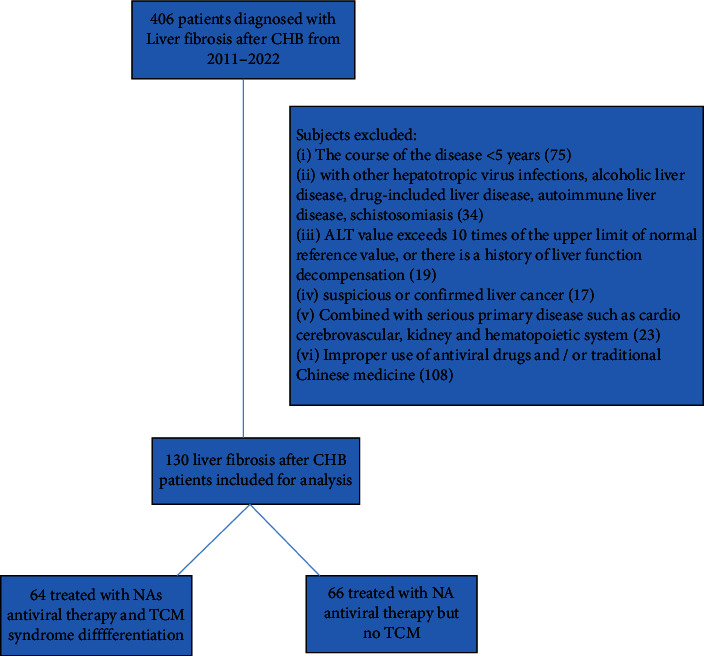
Flowchart of patient recruitment for the study.

A retrospective cohort study was conducted using inpatient and outpatient data sets from 2011 to 2022 in the department of infectious diseases, Longhua Hospital. The data used for analysis include data from the electronic medical record (EMR) and laboratory information systems (LIS), as well as detailed procedures, medication, and TCM prescriptions based on EMR. As shown in Figure 1, taking “liver fibrosis after hepatitis B” as the keyword, a total of 406 patients were screened out, 276 patients of which were excluded from the study.

The diagnostic criteria of the noninvasive liver fibrosis refer to the 2019 guidelines for the diagnosis and treatment of liver fibrosis with integrated traditional Chinese and Western medicine [8], and the inclusion criteria are as follows: (1) Treatment time with NAs ≥1 year, if the recipient of traditional Chinese medicine has been taking it for at least a year. (2) The liver stiffness measurement (LSM, FibroScan) was ≥7.0 kPa [9, 10], abnormal elevation of the serum noninvasive diagnostic model: ARPI ≥ 1 or FIB-4 ≥ 1.45 [11, 12]. (3) TCM diagnosis conforms to the diagnostic criteria for liver fibrosis formulated by the Liver Disease Professional Committee of the Chinese Association of Integrative Medicine [7]. (4) The patients were followed up in Longhua hospital for ≥5 years, and the relevant laboratory indicators of the patients were measured at least once a year during the follow-up period. (5) Major demographic and clinical data are available, such as LSM. There were 130 patients meeting the inclusion criteria finally.

When patients who received TCM for more than 3 months each year were regarded as TCM users, patients who met the inclusion criteria were divided into two groups (TCM users and TCM nonusers), and there are 64 TCM users and 66 TCM nonusers. The details are as follows:

TCM nonusers: NAs monotherapy: entecavir tablets (ETV) 0.5 mg/day or tenofovir disoproxil (TDF) 300 mg/day or tenofovir alafenamide fumarate tablets (TAF) 25 mg/day or lamivudine (LAM) 100 mg/day or adefovir dipivoxil Tablets (ADF) 10 mg/day or LAM 100 mg coupled with ADF10 mg/day. NAs are taken orally every day without interruption.

TCM users: oral NAs combined with TCM. Individualized TCM prescriptions can be taken discontinuously by patients according to the analysis of TCM syndrome distinction, but the total amount of time receiving TCM annually must be more than three months.

### 2.2. Efficacy Evaluation

The main outcome measures were the influence of TCM treatment on liver fibrosis-related indexes, as well as the impact on the occurrence of liver cirrhosis decompensation and/or liver cancer. According to the evaluation standard of the serum noninvasive diagnostic model, the categorization is carried out following with the values of APRI, FIB-4, and LSM. Obvious fibrosis (F2) is defined as 1 ≥ ARPI < 2 or 1.45 ≥ FIB-4 < 3.25 or 7 ≥ LSM < 9.0 kPa; severe fibrosis (F3)/cirrhosis is defined as ARPI ≥ 2 or FIB-4 ≥ 3.25 or LSM ≥ 9.0 kPa. Grading of the data both before and after the treatment, the symptom was improved if the grade in fibrosis decreased. The symptom was steady if there was no change in grade. If the grade increased compared with the prior grade, it meant symptom progression. We compared the changes of HBsAg, HBeAg, and lymphocyte subpopulation after treatment between the two groups to understand the effect of viral immunity on liver fibrosis. The ending occurrences were decided based on the Child's Pugh grade, tumor marker AFP, liver contrast-enhanced CT, or MRI.

### 2.3. Safety Index

Alanine aminotransferase (ALT) and serum creatinine (Scr) were used to evaluate the safety.

### 2.4. Statistical Analyses

Statistical data were described as mean ± standard deviation, median, or percentage. Differences in baseline characteristics between the two groups were assessed by Student's *t-*test or the chi-squared test. If the measurement data obeyed the normal distribution, it was expressed as mean ± standard deviation ( ± *s*). The paired *t-* test was used to compare the data within the group before and after treatment, and the independent sample *t-* test was used to compare the data between the groups. If the normal distribution was not obeyed, the data was expressed by the median (interquartile interval) [*M* (*p*25–*p*75)]. The Wilcoxon signed rank sum test of paired data was used for the comparison of intragroup data before and after treatment, and the Mann–Whitney *U* signed rank sum test of two independent samples was used for the comparison of intergroup data. Pearson was used for comparison of grade data X2-test. The LR positive method of the logistic hazard regression model was used to analyze the independent factors affecting the incidence rate of endind events (decompensated liver cirrhosis or liver cancer). The relative risk (RR) was used to determine whether the exposure factors were related to results. SPSS v25.0 was used for statistical analysis. *P* < 0.05 (bilateral) was considered statistically significant.

## 3. Results

Table 1 describes the baseline characteristics of the patients. There were 49 males and 15 females in the TCM users. The average age was (47.67 ± 10.70) years. The follow-up time ranged from 5 to 15 years, with a median time of 7 years. In the TCM nonusers, there were 41 males and 25 females. The average age was (52.98 ± 10.14) years. The time of follow-up ranged from 5 to 11 years, with a median time of 8 years. There were no statistical differences in baseline characteristics between the two groups, including age, gender, course of disease, smoking history, drinking history, family history, ALT level, HBeAg level, and viral load.

### 3.1. Comparison of Liver Function Indexes between the Two Groups

The Table 2 indicates that AST and TBIL in the TCM users were significantly improved compared with those in the TCM nonusers.

**Figure 2:**
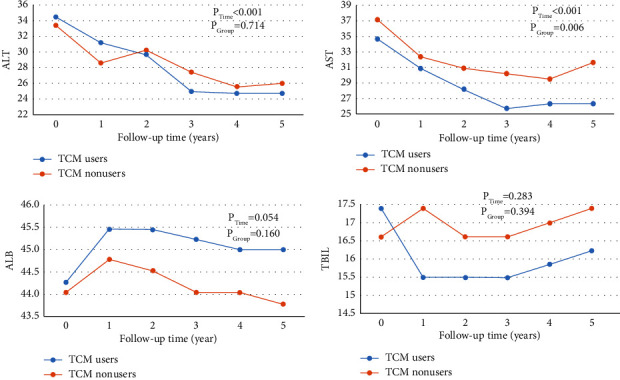
Dynamic observation on the changes of ALB, ALT, AST, and TBIL levels in the two groups during the five-year follow-up. Note: *P*_Time_ indicates the change of liver function indexes with each time point and *P*_Group_ indicates the difference in the subject effect test between the groups.

The date analysis of repeated measurements was performed when we ensured that the data of all patients during the follow-up period met the normality and homogeneity of variance. The dynamic observation of the liver function was managed to perform on 130 patients who were followed for 5 years as shown in Figure 2. It was found that the levels of ALT and AST in the two groups were lower than those before treatment, and the difference of AST between the two groups was statistically significant. There were no statistical differences in ALB and TBIL after treatment compared with before treatment.

According to the results of the time-dependent effect test represented by the follow-up time, there were statistical differences in the levels of ALT and AST at different times (*P*_Time_ < 0.001). The results of the intergroup effect test showed that there were statistical differences in the AST levels between TCM users and TCM nonusers (*P*_Group_=0.006). There was no statistical difference between ALB and TBIL in the test of the time effect and intergroup effect.

### 3.2. Comparison of Noninvasive Liver Fibrosis Indexes between the Two Groups before and after Treatment

**Figure 3:**
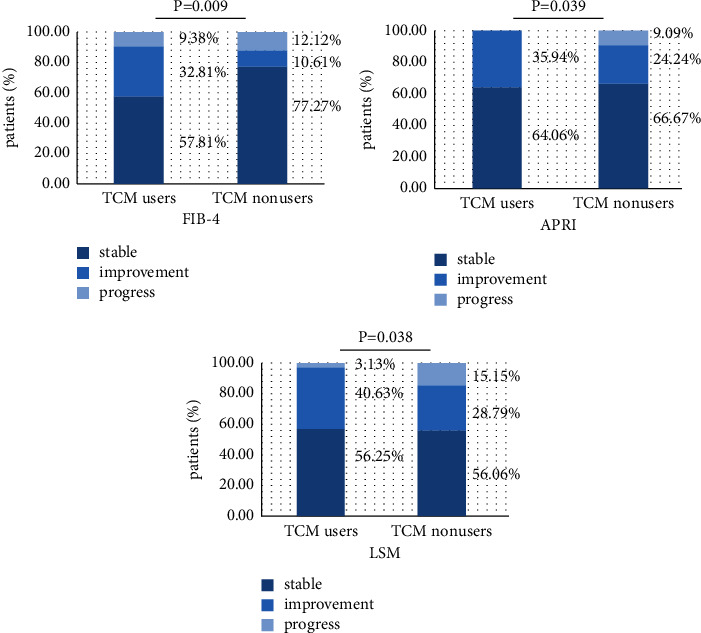
Comparison of FIB-4, APRI, and LSM between the two groups after treatment.

Figure 3 compares the changes of FIB-4, APRI, and LSM between the two groups after treatment. Calculating the two groups' fibrosis grading data, fibrosis was improved in the 40.63% TCM users and 28.79% TCM nonusers according to the LSM (*P*=0.038). Fibrosis was improved in the 32.81% TCM users and 10.61% TCM nonusers in FIB-4(*P*=0.009). Fibrosis was improved in the 35.94% TCM users and 24.24% TCM nonusers in APRI (*P*=0.039).

As shown in Table 3, 37 TCM users and 45 TCM nonusers were compared before and after the treatment finally by excluding the patients with missing four items of the liver fiber during the follow-up period. Because the four items of the liver fiber did not obey the normal distribution, the median (the interquartile interval) was used to represent the baseline. Although there were differences in the median between the two groups, especially the HA level (the normal range of 0–100 ng/ml), there was no statistical difference between the two groups in the baseline characteristics of the four items of liver fiber according to the rank sum test.

Compared to the TCM nonusers, the hyaluronic acid (HA) and collagen type IV (CIV) of TCM users were considerably improved after the treatment. HA decreased significantly from the second year to the fifth year after treatment, and CIV considerably declined between the fourth and fifth years following treatment. Compared with before treatment, the four items of the liver fiber were considerably reduced after TCM treatment. The levels of laminin (LN) and CIV decreased significantly after treatment in TCM nonusers, whereas other items did not change appreciably.

### 3.3. Viral Immunity and Lymphocyte Immune Function

After excluding the patients with incomplete HBV markers during the follow-up, there were 61 patients in the TCM group and 62 patients in the non-TCM group. We observed the changes of HBsAg and HBeAg levels between the two groups (Table 4). When HBsAg level ≥ 250, the level was marked as 1, and HbsAg level < 250, the level was marked as 2. According to the grading changes before and after treatment, it could be divided into improvement, stability, and progress. The results showed that the level of HBsAg in the TCM group was significantly decreased (*P*=0.019), indicating that TCM treatment has a certain effect on reducing the HBsAg level. We marked HBeAg negative as 1 and HBeAg positive as 2, and the HBeAg level in the two groups was observed after treatment. The results showed that there was no significant difference between the two groups (*P*=0.759). Because the sample of HBeAg-positive patients in the 130 patients was too small, the HBeAg negative conversion rate and serum conversion rate were not counted in this study.

As shown in Table 5, the relative counts of CD3+, CD4 +, and CD8+ cells in the TCM users were statistically different compared to those in the TCM nonusers, and CD16+ increased significantly compared to that before treatment. The lymphocyte subpopulation decreased after treatment in the TCM nonusers, and CD3+, CD4+, and CD8+ levels statistically decreased compared with those before treatment.

**Figure 4:**
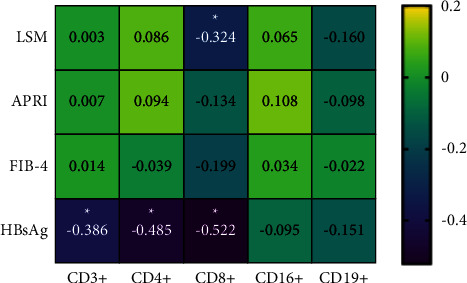
The relationship between fibrosis indexes and the immunological function. Note: ^*∗*^ correlation is significant at the 0.05 level (2-tailed).

As shown in Figure 4, low levels of serum HBsAg were correlated with elevated lymphocyte levels, particularly CD3+, CD4+, and CD8+. Additionally, the high level of CD8+ was significantly correlated with a low level of LSM.

### 3.4. Comparison of PLT, Spleen Thickness, and Length between the Two Groups before and after Treatment

**Figure 5:**
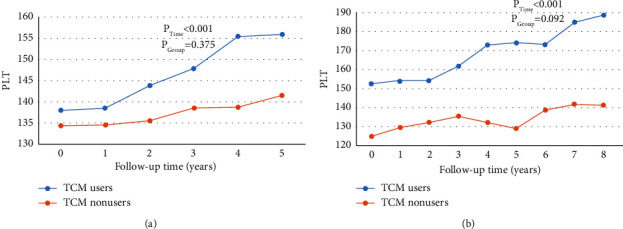
Dynamic observation of the changes of PLT levels in the two groups during the follow-up time. Note: *P*_Time_ indicates the change of PLT with each time point and *P*_Group_ indicates the difference in the subject effect test between the groups. (b) shows that 22 TCM users and 18 TCM nonusers were followed up for 8 years.

Patients with incomplete B-ultrasound data during follow-up were excluded (6 cases in each group). The changes in the spleen and platelet (PLT) between the two groups after treatment are shown in Table 6. The value of PLT in both groups was higher than before treatment, in which the PLT level and spleen thickness of the TCM users were significantly changed compared with those of the TCM nonusers. Meanwhile, the thickness and length of spleen increased significantly after treatment in the TCM nonusers. According to the long-term follow-up observation of the PLT level in Figure 5, the PLT level steadily increased with the prolonging of follow-up time.

### 3.5. Safety Index

As shown in Table 2, there were no significant adverse events, and the ALT of both the groups remained normal during the treatment. The Scr level had no significant difference between the two groups after treatment (Table 7).

### 3.6. Prognosis and Risk

**Figure 6:**
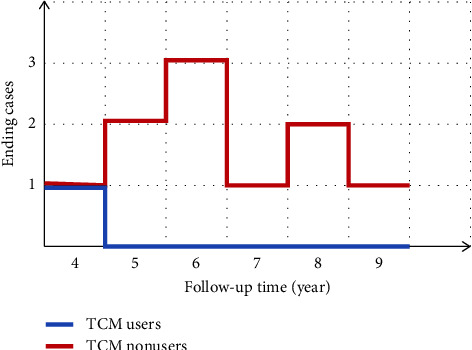
Distribution of the ending in the two groups.

During the follow-up period, there were 12 patients (1 TCM user and 11 nonusers) who suffered liver cancer and/or cirrhosis decompensation. Accordingly, the incidence of ending events increased by 16.67% in the TCM nonusers (compared with 1.56% in the TCM users). The distribution of ending (decompensated cirrhosis and/or liver cancer) in each group is shown in Figure 6. It is noteworthy that there was only one liver cancer patient in the TCM users. As indicated in Table 8, the potential risk factors for the ending were identified and logistic regression analysis was conducted to investigate the risk factors for the ending. The results showed that the *P* value of variable *X*1, *X*10, and *X*11 was less than 0.05, and the difference was statistically significant, suggesting that the course of the disease, family history, and whether to use TCM were the relevant risk factors for the occurrence of ending. According to whether TCM is used as the exposure factor, when the TCM user is in the exposure group, results of the relative risk analysis showed that RR = 0.079 (Table 9), indicating that TCM is beneficial exposure. On the contrary, the RR of disease's course and family history were 9.603 and 21.600, respectively, which were risk factors.

### 3.7. Prescription Analysis

**Figure 7:**
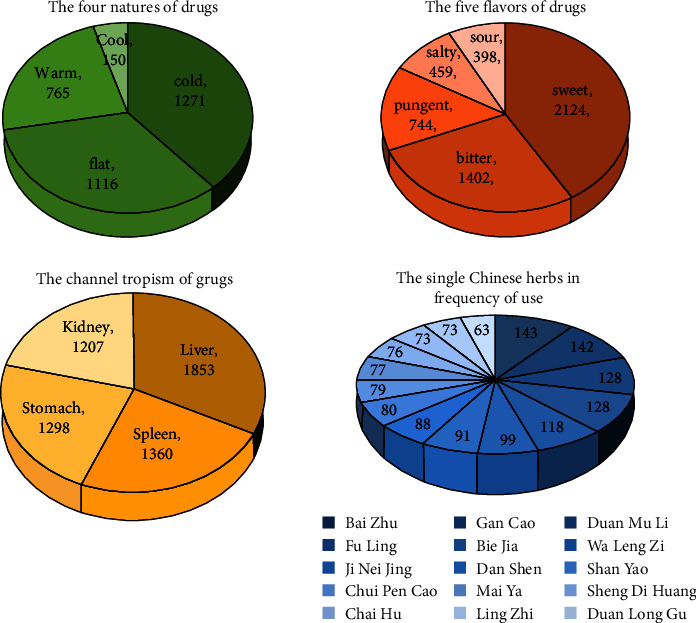
Distribution of the five flavors, four natures, channel tropism, and use frequency of TCM.

A total of 251 single Chinese herbs were collected in the 192 TCM prescriptions from patients with liver fibrosis after CHB. The names and major functions of the most used single Chinese herbs are shown in Table 10, including 15 most commonly prescribed Chinese herbals such as Baizhu (*Atractylodes macrocephala* Koidz), Gancao (*Glycyrrhiza uralensis* Fisch), Fuling (*Poria cocos (Schw.) Wolf*), Duanmuli (*Ostreae Concha*), Biejia (*Carapax Trionycis*), Walengzi (*Carapax Trionycis*), Jineijin (*Endothelium Corneum Gigeriae Galli*), Danshen (*Radix Salviae Miltiorrhizae*), Shanyao (*Rhizoma Dioscoreae Oppositae*), Chuipencao (*SediHerba*), Maiya (*Hordei Fructus Germinatus*), shengdihuang (*Rehmannia Glutinosa Libosch*), Chaihu (*Bupleurum chinense*), Lingzhi (*Citrus reticulata Blanco*), and Duanlonggu (Os Draconis). These medicines have the function of softening hardness and dissipating mass, spleen fortifying, and calming/soothing the liver, which demonstrate that “soften hardness and disperse knots” and “soothe the liver and fortify the spleen” are the main treatment methods of TCM for liver fibrosis after CHB.  Figure 7 reflects the five flavors of TCM, which are primarily bitter and sweet, and drugs' nature are mostly cold and flat, and the channel tropism are mainly the liver and spleen.

## 4. Discussion

The occurrence and development of fibrosis caused by chronic viral hepatitis B are affected by viral factors (such as viral genotype and gene variation, viral load, and viral antigen), host factors (such as immune response, immune escape, cytokines, complement components, and human leukocyte antigen genotype), and their interactions [13]. Hepatitis B virus induces immunosuppressive cells, such as MDSCs, NK-reg, and T-reg cells through an immunosuppressive cascade [14–16]. Excessive immunosuppression may lead to persistent HBV infection, congenital and adaptive immune response dysfunction, liver fibrosis, and HCC progression.

Many clinical studies have shown that TCM has significant advantages in blocking-up liver fibrosis, controlling the development of liver inflammation, and improving clinical symptoms [17]. LSM is accepted as a highly reproducible and accurate indicator for the assessment of liver fibrosis. Therefore, FibroScan was used to track the improvement in liver stiffness brought on by TCM, and the results showed that the rate of improvement after treatment was 40.63%, which was much better than the 28.79% of the TCM nonusers. APRI and FIB-4 are relatively mature serum noninvasive detection models, in which the sensitivity and specificity of APRI in evaluating liver fibrosis after hepatitis B are 0.87 and 0.66 [18] and of FIB-4 are 0.78 and 0.65 [19], respectively. According to results of our research, both of them effectively predicted the improvement of TCM on liver fibrosis.

By analyzing the four items of liver fibrosis, we found that HA and CIV in patients receiving TCM together with NAs were statistically considerably better than the TCM nonusers. Existing research studies have found that elevated serum Col IV is related to degrees of fibrosis and severity of hepatitis, and it is served as a histochemical marker of perisinusoidal basement membrane formation in liver disease [20]. Most researchers have proved that there is a good correlation between serum hyaluronic acid levels and liver fibrosis development [21], and it can be considered as a reliable noninvasive glycoprotein for discriminating the patients with liver fibrosis from the healthy ones to assess the liver fibrosis grade [22].

The AST level is one of the independent predictors of significant fibrosis [11], which was used in the liver fibrosis noninvasive diagnostic model of APRI and FIB-4. The serum bilirubin level represents hepatic synthetic and excretory function, which is the component of most well-recognized prognostic models including the –Child–Pugh score and model for end-stage liver disease (MELD) score [23]. Our data shows that AST and TBIL after TCM treatment are significantly lower than those in the TCM nonusers, and AST levels are different between the different groups at each time point of follow-up, suggesting that AST and TBIL may also predict the severity and long-term prognosis of patients with chronic hepatitis B liver fibrosis.

In patients with CHB, HBsAg plays an important role in predicting the level of liver fibrosis in HbeAg positive and HbeAg negative patients [24]. However, when CHB is established, HbsAg serum clearance is rarely observed, which may be due to the impairment of innate and adaptive immunity and the exhaustion of T-cell and B-cell responses [25]. The activation of antiviral T cells contributes to the decline of HbsAg [24]. Our results showed that the majority of TCM users' serum HbsAg levels decreased after treatment, while the increase of lymphocyte CD3+, CD4+, and CD8+ was significantly correlated with low-level HbsAg. These results indicate that TCM combined with antiviral drug NAs can help maintain a low baseline of HbsAg and HBV DNA by improving lymphocyte levels.

An experimental research has suggested that fibrosis induced by acute HBV infection leads to the immune disorder of intrahepatic lymphocytes and the immune function is inhibited [26]. As hepatitis B virus replication enters the chronic phase, it is characterized by quantitatively and qualitatively weak HBV-specific CD8+ T-cell responses. The progress of liver fibrosis during HBV infection may also jeopardize hepatocellular antigen recognition by intravascular CD8+ T cells when crawling along liver sinusoids [27]. Meanwhile, the presence of fibrosis/cirrhosis was also associated with a lower B cell allostimulatory capacity, manifested in the reduced ability of alloreactive CD4+ T cells producing the antifibrotic factor after the B-cell contact [28]. The number of CD3+, CD4+, and CD8+ in patients treated with TCM was significantly higher than that in TCM nonusers, and there was a negative correlation between CD8+ and the noninvasive fibrosis index (LSM), which indicates that TCM has a definite impact on restoring and enhancing the lymphocyte immune function and inhibiting fibrosis.

According to the TCM theory, liver fibrosis after CHB is caused by HBV invading the body and being alive for a long time. In addition, improper diet, heavy labor, unsmooth mood, insufficient healthy qi, etc. can damage the immune function. Besides, the virus stays in the liver for a long time, so the liver cannot work normally. The disease location is mainly related to the liver, and the spleen, stomach, and kidney also are involved [29], which is consistent with our statistical results (Figure 7). In terms of treatment, the consensus believes that liver diseases were caused by “upright weakness” and “blood stasis” [7]. The prescription data demonstrates that the single herbs with a frequency of more than 50% are mainly softening-hardness and stasis-eliminating medicinal such as *Ostreae concha*, *Carapax trionycis*, *Arcae concha*, and *Radix Salviae Miltiorrhizae*, and spleen-fortifying medicinal such as *Atractylodes macrocephala*, *Glycyrrhiza uralensis fisch*, and *Poria cocos*; the latter helps to improve patients' own immunity and reduce the continuous harm of the virus to the body.

In modern pharmacological research, they mainly play the role of anti-inflammatory agents and liver protection, inhibiting the activation of hepatic stellate cells, reducing liver fibrosis, regulating immunity, and enhancing the lymphocyte function. For example, licorice total flavonoids, a natural source of hepatoprotective activity, can heal liver tissue and reduce hepatic injury via alleviating inflammation, improving the antioxidant enzyme activity, and reducing oxidative stress in liver tissue [30]. The hepatoprotective effects of Poria cocos polysaccharides are related to the molecular mechanisms of suppressing cell death, reducing hepatocellular inflammatory stress, and heat shock protein bioactivity [31]. Both the extract and pure peptides of *Carapax trionycis* display strong hepatoprotective effects. Among them, oligopeptide I-C-F-6 has been proved to have a antifibrosis effect in the animal model of liver fibrosis, the mechanism of which is related to modulating NF-kB and Wnt/b-catenin signaling [32]. In immunocompromised mice, oyster oligopeptide could significantly enhance the proliferation of spleen lymphocytes, NK cell activity, regulate the distribution of CD4+T and CD8+T spleen lymphocyte subsets, and positively regulate the cell immunity, humoral immunity, and nonspecific immune function [33]. Oyster peptide can restore the index of the thymus, spleen, and liver, stimulate cytokine secretion, and affect the occurrence and duration of chronic hepatitis B by regulating the balance of Th1/Th2 [34].

There are limitations in this study. Firstly, because it is not a randomized and controlled trial, there may be a potential bias due to the influence of economy, work, and family environment. Some patients choose different oral methods of TCM preparations, which will have different efficacy, so a further research is needed. Secondly, due to the limitation of detection technology, HBV DNA cannot detect the amount of virus below 100 IU/ml, and it is impossible to determine whether the virus is completely suppressed. Thirdly, TCM syndrome differentiation is mainly based on the personal experience of doctors. When patients choose different doctors, it may lead to different tendencies of four natures and five flavors of TCM in the prescription. Finally, our research data are limited because the patients mainly come from Longhua Hospital, so a multicenter study should be conducted to verify the efficacy of traditional Chinese medicine.

## 5. Conclusions

In conclusion, this retrospective study indicates that TCM combined with NAs in the treatment of patients with liver fibrosis after chronic hepatitis B can significantly improve fibrosis indicators, stabilize the liver function, maintain a low baseline of HBsAg and HBVDNA, promote the recovery of patients' immune system, and help block or even regress the process of liver fibrosis in patients with chronic hepatitis B.

## Figures and Tables

**Table 1:** Baseline characteristics of two groups.

Characteristics	TCM users (*n* = 64)	TCM nonusers (*n* = 66)	*P* value
*Age (years)*			
<40	11 (17.2%)	7 (10.6%)	0.277
≥40	53 (82.8%)	59 (89.4)	

*Gender*			
Male	49	41	0.074
Female	15	25	

*Duration of illness*			
5∼10	53	50	0.322
10∼	11	16	

*Smoking history (n, %)*			
Yes	2	1	0.978
No	62	65	

*Drinking history (n, %)*			
Yes	0	2	0.490
No	64	64	

*Family history of CHB-related diseases (n, %)*			
Yes	3	9	0.078
No	61	57	

*HBsAg*			
<250	15	22	0.211
≥250	49	44	

*HBeAg*			
Positive	23	16	0.146
Negative	41	50	

*HBV DNA level (copies/mL)*			
<10^2^	50	54	0.871
10^2^∼10^3^	7	6	
10^3^∼10^4^	7	6	

*ALT (IU/ml ULN)*			
1∼2	45	54	0.124
2∼3	19	12	

*LSM*			
7∼9	12	21	0.087
≥9	52	45	

**Table 2:** Comparison of liver function indexes between the two groups before and after the treatment [*M* (*p*25–*p*75), $\bar{x}$ ± *s*].

	Treatments	ALB	ALT (U/L)	AST (U/L)	TBIL (*μ*mol/L)
TCM users	Before	43.35 ± 4.92	36.92 ± 19.08	37.00 (28.00–50.75)	16.50 (12.50–21.78)
	After	43.73 ± 3.73	25.83 ± 13.10^*∗*^	25.00 (21.25–31.75)^*∗*^^△^	15.70 (12.50–21.83)^△^

TCM nonusers	Before	43.05 ± 5.25	34.94 ± 14.76	37.50 (28.00–49.00)	16.10 (12.48–22.25)
	After	42.25 ± 4.54	27.88 ± 13.62^*∗*^	29.00 (24.00–38.25)^*∗*^	18.25 (14.60–22.18)^*∗*^

Note. Compared with that before treatment in this group, ^*∗*^*P*  <  0.05; compared with the TCM nonusers after treatment, ^△^*P*  <  0.05.

**Table 3:** Comparison of four items of the liver fiber before treatment [*M* (*P*25–*P*75)].

Years	Groups	HA (ng/ml)	PCIII (ng/ml)	CIV (ng/ml)	LN (ng/ml)
0	TCM users	83.40 (57.40–206.30)	13.90 (6.40–41.75)	62.50 (43.80–69.35)	78.50 (44.55–114.20)
	TCM nonusers	118.70 (64.20–194.95)	9.70 (6.05–23.95)	65.60 (33.20–100.90)	79.10 (46.30–106.90)
	*P* value	0.240	0.612	0.228	0.741

1	TCM users	89.60 (62.05–176.05)	6.70 (5.00–23.65)	58.40 (41.65–70.85)	68.40 (40.72–100.60)
	TCM nonusers	160.90 (89.10–268.35)	8.30 (5.15–55.60)	63.20 (31.40–118.95)	58.80 (38.00–88.25)
	*P* value	0.190	0.172	0.071	0.096

2	TCM users	114.50 (95.70–129.40)	12.30 (5.80–43.85)	48.10 (35.20–69.60)	67.30 (44.70–96.48)
	TCM nonusers	131.20 (116.70–144.00)	7.30 (5.10–18.90)	47.70 (33.70–64.05)	57.90 (35.85–98.1)
	*P* value	0.442	0.154	0.001	0.655

3	TCM users	77.10 (63.15–87.00)	9.00 (5.45–18.20)	35.30 (20.40–35.30)	36.44 (24.15–52.98)
	TCM nonusers	130.10 (119.30–149.25)	6.90 (4.30–13.45)	40.01 (23.40–91.90)	50.50 (22.60–77.79)
	*P* value	0.084	0.131	0.000	0.228

4	TCM users	60.90 (58.45–97.50)	13.90(5.50–18.60)	26.90(16.85–44.45)	23.50 (15.10–57.05)
	TCM nonusers	124.40 (114.45–137.20)	12.30 (5.20–18.30)	36.80 (19.55–92.55)	34.80 (22.55–54.20)
	*P* value	0.020	0.499	0.000	0.208

5	TCM users	67.10 (58.95–96.50)^*∗*^^△^	10.10 (5.25-17.20)^*∗*^	24.90 (14.90–35.20)^*∗*^^△^	39.50 (22.35–72.90)^*∗*^
	TCM nonusers	106.00 (69.9–170.25)	12.40 (6.45–19.55)	32.80 (19.10–71.45)^*∗*^	39.50 (22.35–72.90)^*∗*^
	*P* value	0.008	0.207	0.001	0.053

Note. Compared with that before treatment (0 year) in this group, ^*∗*^*P*  <  0.05; compared with the TCM nonusers after treatment, ^△^*P*  <  0.05.

**Table 4:** Comparison of HBsAg and HBeAg between the two groups after treatment.

	Groups	Improvement	Stable	Progress	Total	Mean rank	*Z*	*P*value
HBsAg	TCM users	4	55	2	61	56.84	−2.344	0.019
	TCM nonusers	4	45	13	62	67.07		

HBeAg	TCM users	12	48	1	61	61.33	−0.3070	0.759
	TCM nonusers	10	52	0	62	62.66		

**Table 5:** Comparison of lymphocyte subpopulation between the two groups [$\bar{x}$ ± *s*].

	Cases	Treatments	CD3+ (%)	CD4+ (%)	CD8+ (%)	CD16+ (%)	CD19+ (%)
TCM users	24	Before	71.13 ± 11.71	44.03 ± 10.67	25.28 ± 8.70	16.15 ± 12.03	11.98 ± 5.39
		After	71.59 ± 9.68^△^	42.61 ± 6.83^△^	25.01 ± 8.22^△^	19.44 ± 10.41^*∗*^	11.20 ± 4.12

TCM nonusers	29	Before	73.47 ± 9.65	49.20 ± 9.94	26.29 ± 8.03	18.64 ± 9.56	14.16 ± 6.56
		After	62.05 ± 10.7^*∗*^	38.29 ± 8.29^*∗*^	18.92 ± 5.64^*∗*^	17.72 ± 8.48	13.07 ± 4.55

Note. Compared with that before treatment in this group, ^*∗*^*P* < 0.05; compared with the TCM nonusers after treatment, ^△^*P* < 0.05.

**Table 6:** Comparison of PLT, spleen thickness, and length between the two groups.

	Treatments	PLT (10^9^/L)	Spleen thickness (mm)	Spleen length (mm)
TCM users	Before	84.00 (84.00–180.00)	35.00 (30.00, 44.25)	100.00 (92.75, 125.25)
	After	149.50 (105.50–224.50)^*∗*^^△^	36.50 (30.00, 41.25)^△^	106.50 (90.75, 120.00)

TCM non-users	Before	124.00 (75.75–176.25)	36.50 (30.00, 45.00)	104.00 (89.25, 125.75)
	After	134.00 (95.25–177.25)	40.50 (31.25, 46.00)^*∗*^	111.00 (90.25, 128.75)^*∗*^

Note. Compared with that before treatment in this group, ^*∗*^*P* < 0. 05; compared with the control group after treatment, ^△^*P* < 0. 05.

**Table 7:** Comparison of Scr between the two groups before and after treatment [$\bar{x}$ ± *s*].

	Cases	Scr (*μ*mol/l, *x* ± *s*)	
		Before treatment	After treatment
TCM users	64	65.15 ± 12.32	71.19 ± 14.91^*∗*^
TCM nonusers	66	65.68 ± 14.03	67.41 ± 16.40

Note. Compared with that before treatment in this group, ^*∗*^*P* < 0 05; compared with the control group after treatment, ^△^*P* < 0.05.

**Table 8:** Logistic regression analysis of the influence of some risk factors on ending.

Factors	Variable names	Assignment description
Course of disease (years)	*X*1	<7 = 1, ≥7 = 2
Age (years)	*X*2	<40 = 1, 40∼49 = 2, 50∼59 = 3, 60∼69 = 4, 70∼80 = 5
Gender	*X*3	Male = 1, female = 2
HBsAg	*X*4	<250 = 1, ≥250 = 2
HBeAg	*X*5	Negative = 0, positive = 1
HBV DNA level	*X*6	∼10^2^ = 1, ∼10^3^ = 2, 10^3^∼ = 3
Other basic diseases	*X*7	No = 0, yes = 1
Smoking	*X*8	No = 0, yes = 1
Drinking	*X*9	No = 0, yes = 1
Family history	*X*10	No = 0, yes = 1
TCM use or not	*X*11	No = 0, yes = 1
Ending^*∗*^	*Y*	No = 0, yes = 1

^*∗*^indicates whether the outcome is decompensated cirrhosis and/or liver cancer.

**Table 9:** Estimated values for independent variables and important parameters in the equation.

Variable names	B	S.E	Wald	Df	Sig.	Exp (B)	95% CI
*X*1	2.262	1.061	4.549	1	0.033	9.603	(1.201, 76.778)
*X*10	3.073	0.696	19.497	1	0.000	21.600	(5.522, 84.488)
*X*11	−2.534	1.061	5.707	1	0.017	0.079	(0.100, 0.635)

Note. Dependent variable: *Y* (liver cancer/decompensation); independent variables: *X*1 (course of disease), *X*10 (family history), and *X*11 (TCM use or not).

**Table 10:** The names and major functions of the single Chinese herb medicinal mostly used in TCM prescriptions for liver fibrosis after CHB.

Chinese herb medicines	Usual doses (g)	Major functions	No. of users	(%)
*Atractylodes macrocephala Koidz*. (Bai Shu)	12	Fortifies the spleen and replenishes qi, dries dampness, and induces diuresis	143	74.19
*Glycyrrhiza uralensis Fisch* (Gan Cao)	6	Fortifies the spleen and replenishes qi, clears heat and detoxifies, dispels phlegm to suppress cough, relieves spasm and pain, and moderate herbs	142	73.67
*Poria cocos* (schw.) Wolf (Fu Ling)	9	Induces diuresis to dry dampness, fortifies the spleen, and tranquilizes the heart	128	66.41
*Ostreae Concha* (Duan Mu li)	15	Softens the hardness and disperses the knot, calms the liver and hides the yang, calms the nerves, astringency, restrains acid, and relieves pain	128	66.41
*Carapax Trionycis* (Bie Jia)	12	Nourishes yin and yang, reduces fever and steam, softens hardness, and disperses knot	118	61.22
*Arcae Concha* (Wa Leng Zi)	15	Eliminates phlegm, softens hardness, removes blood stasis and disperses stagnation, restrains acid, and relieves pain	99	51.36
*Endothelium Corneum Gigeriae Galli* (Ji Nei Jin)	12	Eliminates food, strengthens the stomach, and astringes the essence	91	47.21
*Radix Salviae Miltiorrhizae* (Dan Shen)	9	Promotes blood circulation and removes blood stasis, relieves pain, clears heat, and cools blood	88	45.66
*Rhizoma Dioscoreae Oppositae* (Shan Yao)	15	Invigorates the spleen and stomach, generates fluid and benefits the lung, tonifies the kidney, and astringent the essence	80	41.51
SediHerba (Chui Pen Cao)	12	Removing dampness and jaundice and clearing away heat and toxin	79	40.99
*Hordei Fructus Germinatus* (Mai Ya)	12	Invigorating qi and spleen, digestion, soothing liver, and regulating qi	77	39.95
*Rehmannia Glutinosa Libosch* (Sheng Di Huang)	12	Clearing heat and cooling blood, nourishing yin, and generating body fluid	76	39.43
*Bupleurum chinense* (Chai Hu)	6	Harmonizes and releases the exterior and interior, soothes the liver to relieve depression, raises yang and lifts prolapsed organs, and interrupts malaria	73	37.87
*Citrus reticulata Blanco* (Ling Zhi)	9	Replenishes qi, calms nerves, and relieves cough and asthma	73	37.87
Os Draconis (Duan Long Gu)	15	Calms the nerves, calms the liver and hides the yang, and astringency	63	32.69

## Data Availability

In order to protect patient's privacy, we only provide datasets to the editor or editorial staff.

## Abbreviations

CHB: Chronic hepatitis B
ALB: Albumin
ALT: Almandine aminotransferase
AST: Aspartate aminotransferase
GGT: *γ*-Glutamyl transpeptidase
TBil: Total bilirubin
PLT: Platelet
Scr: Serum creatinine
LSM: Liver stiffness measurement
95% CI: 95% confidence interval
HA: Hyaluronic acid
CIV: Collagen type IV
PCIII: Procollagen type III
LN: Laminin.
